# Management of Multisystem Inflammatory Syndrome in Children (MIS-C) in resource limited settings: The Kenyan Experience

**DOI:** 10.21203/rs.3.rs-1951206/v1

**Published:** 2022-08-17

**Authors:** ANGELA NYANGORE MIGOWA, Pauline Samia, Jasmit Shah, Sean del Rossi, Chemutai Kenei, Oliver Ombeva Malande, Joy Ayaya, Daisy Jeruto, Laura Oyiengo, Laura Lewandowski

**Affiliations:** Aga Khan University Medical College East Africa; Aga Khan University Medical College East Africa; Aga Khan University Medical College East Africa; Aga Khan University Medical College East Africa; University of Nairobi College of Health Sciences: University of Nairobi Faculty of Health Sciences; Nakuru Provincial General Hospital; Maseno University; Kabarak University; UNICEF Eastern and Southern Africa Regional Office; NIAMS: National Institute of Arthritis and Musculoskeletal and Skin Diseases

## Abstract

**Background:**

Since the onset of the recent COVID-19 pandemic, there have been growing concerns regarding multisystem inflammatory syndrome in children (MIS-C). This study aims to describe the clinico-epidemiological profile and challenges in management of MIS-C in low-middle income countries by highlighting the Kenyan experience.

**Methods:**

A retrospective study at the Aga Khan University Hospital Nairobi, Avenue Hospital Kisumu and Kapsabet County Referral Hospital was undertaken to identify cases of MIS-C. A detailed chart review using the World Health Organization (WHO) data collection tool was adapted to incorporate information on socio-demographic details and treatment regimens.

**Findings:**

Twenty children with MIS-C were identified across the three facilities. Seventy percent of the children were male (14 of 20). COVID-19 PCR testing was done for five children and only one was positive. The commonest clinical symptoms were fever (90%), tachycardia (80%), prolonged capillary refill (80%), oral mucosal changes (65%) and peripheral cutaneous inflammation (50%). Four children required admission into the critical care unit for ventilation support and inotropic support. Cardiac evaluation was available for six patients four of whom had myocardial dysfunction, three had valvulitis and one had pericarditis. Immunoglobulin therapy was availed to two children and systemic steroids provided for three children. There were no documented mortalities.

**Interpretation:**

We describe the first case series of MIS-C in East and Central Africa. Majority of suspected cases of MIS-C did not have access to timely COVID-19 PCR testing and other appropriate evaluations which highlights the iniquity in access to diagnostics and treatment.

## Introduction

The coronavirus disease 2019 (COVID-19) caused by severe acute respiratory syndrome coronavirus 2 (SARS-CoV-2) has led to the ongoing pandemic afflicting individuals of all ages (1) with over six million cases diagnosed globally (2). Though children are less likely than adults to become severely ill, preschool-aged children and infants are more likely than older children to have severe clinical manifestations (2–4).

Approximately a quarter of all COVID-19 positive children have underlying diseases such as asthma, cardiovascular disease and immunosuppressive conditions (3). Dong et al in China noted that children of all ages appeared susceptible to COVID-19, without any significant gender difference (5). Although clinical manifestations of children’s COVID-19 cases were generally less severe than those of adult patients, young children, particularly infants, were vulnerable to infection (5).

MIS-C is a novel syndrome among children and adolescents which includes a multi-system inflammatory condition similar to Kawasaki disease and toxic shock syndrome (1, 4, 6). It is postulated that the COVID-19 virus causes capillary inflammation with complement activation (7, 8). This acute presentation leads to multi-organ failure and shock (1, 4, 6). There is now a growing number of children presenting with a multisystem inflammatory syndrome (8). Features that may distinguish MIS-C from Kawasaki syndrome include prominent cardiac dysfunction, severe enteropathy and relative thrombocytopenia (11). MIS-C may also present with peculiar abdominal symptoms and elevated inflammatory markers (8). Laboratory testing has detected positive serology in majority of patients linking COVID-19 as a possible cause of this presentation (1, 4). Children have responded to anti-inflammatory treatments, including parenteral immunoglobulin, steroids and biological therapies (1, 9, 10).

Given that MIS-C is a rare but potentially fatal condition, more data is required to understand the differences in burden, severity and outcomes of COVID-19 in low-middle income countries compared to high-income countries. It is important to describe the clinical features and outcomes of MIS-C in our context within sub-Sahara Africa. Better characterization of MIS-C will be useful in defining optimal management approaches applicable within our region (11) (12).

Assessment of the rates and long-term effects of MIS-C on children will be important in accurately modelling the pandemic and to ensure that appropriate resources are allocated to children requiring care (2). Due to the vaccine inequity between high income and low income countries, children in Kenya and similar settings are likely to lag behind in vaccination thus putting them at higher risk of developing MIS-C. Furthermore, research has shown that clinical outcomes of MIS-C among black children are worse compared to other races (13). Consequently, forecasting resources for COVID-19 alone without adequately planning for possible MIS-C that may follow childhood infections would be inadequate.

The COVID-19 pandemic is still evolving and the impact of this disease particularly in the paediatric population is still largely unknown. The data obtained from this study shall contribute towards a better understanding of the MIS-C presentation in the COVID-19 era, response to available therapies and prognosis among these children in low middle income countries.

## Methods

### Study setting

We carried out a retrospective cohort study between 1st August 2020 to 31st August 2021. Charts of paediatric inpatients were reviewed at three facilities; the Aga Khan University Hospital, Nairobi (AKUHN), Avenue Hospital in Kisumu County and Kapsabet County Referral Hospital in Nandi County.

### Inclusion Criteria

Data from medical records of all patients aged 0–18 years who fulfilled the World Health Organisation (WHO) MIS-C case definition; (16) with fever (body core temperature of at least 38C or more) lasting more than three days

AND two of the following:

1. Rash or bilateral non-purulent conjunctivitis or muco-cutaneous inflammation signs (oral, hands or feet).
2. Hypotension or shock.
3. Features of myocardial dysfunction, pericarditis, valvulitis, or coronary abnormalities (including ECHO findings or elevated Troponin/NT-proBNP),
4. Evidence of coagulopathy (by PT, PTT, elevated d-Dimers).
5. Acute gastrointestinal problems (diarrhoea, vomiting, or abdominal pain).

AND elevated markers of inflammation such as ESR, C-reactive protein, or procalcitonin AND no other obvious microbial cause of inflammation, including bacterial sepsis, staphylococcal or streptococcal shock syndromes AND evidence of COVID-19 (RT-PCR, antigen test or serology positive), or likely contact with patients with COVID-19.

Contacts with COVID - 19 cases were defined as follows;

1. A child living in the same household as a COVID-19 case;
2. A child having had direct physical contact with a COVID-19 case (e.g. shaking hands);
3. A child having unprotected direct contact with infectious secretions of a COVID-19 case (e.g. being coughed on, touching used paper tissues with a bare hand);
4. A child having had face-to-face contact with a COVID-19 case within 2 metres and for more than 15 minutes;
5. A child who was in a closed environment (e.g. classroom, meeting room, hospital waiting room, etc.) with a COVID-19 case for 15 minutes or more and at a distance of less than 2 metres;
6. A healthcare worker (HCW) or other person providing direct care for a COVID-19 case, or laboratory workers handling specimens from a COVID-19 case without recommended personal protective equipment (PPE) or with a possible breach of PPE.
7. A contact in an aircraft sitting within two seats (in any direction) of the COVID-19 case, travel companions or persons providing care, and crew members serving in the section of the aircraft where the index case was seated (if severity of symptoms or movement of the case indicate more extensive exposure, passengers seated in the entire section or all passengers on the aircraft may be considered close contacts).
8. A contact in a public service vehicle where it cannot be confirmed that the sitting arrangement is as per government requirements.

Data from the following patients were excluded from the study; children with known congenital or acquired cardiac disease, children with known gastro-intestinal disease and children already known to have autoimmune and auto-inflammatory conditions.

### Study Methods

Approvals for this study were obtained from the Institutional Ethics Review Committee at the Aga Khan University *(Ref: 2020/IERC-86(v2),* the National Commission for Science, Technology and Innovation-NACOSTI *(NACOSTI/P/20/6232)* and Ministry of Health of Kenya for the participation of the respective institutions.

The study investigators held online workshops with lead clinicians to review the case identification process, the data collection sheet and documentation procedures to ensure uniform recruitment procedures across study centres.

Medical records of both paediatric inpatients and outpatients who fulfilled the WHO MIS-C case definition underwent a detailed chart review by a trained recruiter at the health facility and data captured using an adapted WHO data collection tool collected and securely stored on the Research Electronic Data Capture - REDCap platform (Vanderbilt and National Institute of Health) (14). Additional information was collected including education and economic status of the child’s legal guardian, information on drug name, dose, route, frequency, duration and adverse effects noted during administration of these drugs to the patients.

Following data collection, records were cross checked for completeness and accuracy. All patient data was de-identified to ensure confidentiality and unique identifier utilized for data entry into REDCap. In order to minimize chances of missing data, the online REDCap forms had prompts that prevented progress if fields were missing data when information is required and also reminders to check that all information was entered correctly.

Descriptive statistics were presented where categorical data was presented as frequencies and percentages while continuous data was presented as medians and interquartile ranges. SPSS was used for analysis (IBM Statistical Package for the Social Sciences version 22·00).

## Results

### Demographics

Twenty children were identified to have MIS-C in this study. Among the 47 county regions approached to participate in this study, only three counties identified MIS-C cases from the period August 2020 to August 2021 with 60% of the cases (12 of 20) identified within Nairobi County. Seventy percent (14 of 20) were male and 85% (17 of 20) were of African ancestry. The median age at diagnosis was 3·98 years (range 1·71 – 7·44). Among participants enrolled, 75% (15 of 20) had access to health insurance to help cater for their medical costs.

### Clinical Features of MIS-C Patients

Thirty-five percent of MIS-C patients (7 of 20) had respiratory symptoms 4 weeks prior to the onset of MIS-C. Only 10% of the patients (2 of 20) reported a confirmed history of COVID-19, two (10%) had household members with confirmed COVID-19 infection, 6 (30%) had contact with COVID-19 household members. Fifty percent (10 of 20) of the participants did not have COVID-19 test done or any known history of COVID-19 infection nor contact but suspected to have community exposure. Twenty-five per cent of the children (5 of 20) had a COVID-19 test done of which only one had a positive PCR test and another had a positive antibody test. All the five children tested had a negative COVID - 19 antigen test. None of the children had received prior COVID-19 vaccinations.

Fever was the most common clinical feature, present in 90% (18 of 20) of the children at admission. Eighty percent (16 of 20) presented with tachycardia and prolonged capillary refill time. Vomiting was observed among 75% of the children (15 of 20) and 50% (10 of 20) had abdominal pain and diarrhea. Table 1 illustrates the clinical features of the patients at admission. As regards laboratory investigations, inflammatory markers were elevated with a relatively low neutrophil count. Table 2 below illustrates the laboratory features of the patients at admission.

### Severe Organ System Involvement

Four of the 20 patients with MIS-C, required critical care with ventilation support. One patient required inotropic support.

### Cardiac Complications

Echocardiogram evaluation was done for six children and revealed four had myocardial dysfunction, three had valvulitis and one had pericarditis. There was no documentation of any further follow up except for one child who had normal cardiac evaluation at six weeks post discharge. There were no mortalities documented during the period of the study.

## Discussion

We report 20 MIS-C patients cross 3 centers in Kenya. The median age was 3·98 years (range 1·71 – 7·44) and 70% of the children were male. Our colleagues in South Africa had a cohort of 68 patients with a much higher median age of 7 years (range 3·6–9·9) (15). The majority of our patients were African, reflecting the demographics of our nation.

In our cohort, MIS-C was distinguished by certain demographic features and clinical presentations including being aged 6 to 12 years, fever (90%), tachycardia (80%), prolonged capillary refill (80%), vomiting (75%), oral mucosal changes (65%) and peripheral cutaneous inflammation (50%). Similarly, in the South African study by Webb and co-workers, the most common clinical features in children with MIS-C were fever (100%), tachycardia (98·5%), rash (85·3%), conjunctivitis (77·9%), abdominal pain (60·3%) and hypotension (60·3%) (15). Eighty five percent of the children in the South African cohort had gastrointestinal symptoms, including abdominal pain (60·3%), diarrhea (58·8%) and vomiting (10/41) (15).

In the cohort studied by Webb and colleagues, twenty-three percent of children with MIS-C (14/61) had a confirmed SARS-CoV-2 contact, 18% (11/61) had a suspected contact and 59% (36/61) had no contact reported (15). In our cohort only 10% of the patients (2 of 20) reported a confirmed history of COVID-19, 2 (10%) had household members with confirmed COVID-19 infection, six (30%) had household members with suspected COVID 19 infection. The other 10 participants (50%) did not have COVID 19 test done or any known history of COVID 19 infection nor contact. It is thus important to highlight children exposed to COVID-19 infection can be severely ill with negative COVID-19 tests and hence a high index of suspicion is key in detecting MIS-C cases. In addition, lack of diagnostics in our context might have contributed to the low testing and positivity rate.

There are few reports of MIS-C is sub-Saharan Africa, which may lead to a presumption that this syndrome is not identified in these populations yet low and middle-income countries (LMICs) have the challenge of delayed vaccination, poor vaccine coverage, inadequate diagnostics, sub-optimal management options despite a relatively larger proportion of younger people in their population (15).

In every cohort reported to date, black children are disproportionately affected by MIS-C (1, 2, 13, 14) with an estimation of a nearly six times higher risk of developing MIS-C as compared to white children (15). In the case series from South Africa by Webb and colleagues for example, they found that black children were over-represented in the MIS-C group (62% vs 37%, p = 0·002) (15). Kenya is a low-middle income country (LMIC), but the centers reporting cases had the resources to identify, treat and document these cases. In rural areas, it is possible that more children remain undiagnosed due to lack of resources and this may explain the low or no detection rate in other county referral hospitals across Kenya further highlighting inequities in resources available to manage these patients.

Work by Feldstein revealed presenting symptoms and signs were similar among patients with MIS-C and COVID-19 with the exception of muco-cutaneous findings (66·8% [95%CI, 63%−71 %] vs 10·2% [95%CI, 8%−13%]; p < .001), which were uncommon in patients with COVID-19 and prevalent in those with MIS-C (16). One fifth of the patients in their cohort required critical care support (16). Similar findings were found by Odisha and colleagues in eastern state of India that analyzed a cohort of 21 children the majority of whom were male (76·2%) and the predominant age group was 6–10 years (16). However, unlike our cohort which had no mortality reported, Odisha and co-workers reported a mortality of 9% in their cohort which is higher than that reported in western literature (16). Majority of their cases were positive for severe acute respiratory syndrome coronavirus antibody (16). Solanki and co-workers in eastern India studied a cohort of 10 children and there was no mortality; findings which are similar to our cohort (17). Further studies are required to help predict which category of children are likely to develop MIS-C and progress on to have the risk of mortality (17). This will help us prioritize resources for the paediatric population that truly need it.

As regards laboratory investigations, our cohort revealed patients with MIS-C had markedly elevated inflammatory markers. It is important to note that less than 50% of our cases had the laboratory investigations of interest such as inflammatory markers, white cell count, platelet count, INR, ferritin and electrolytes. In the cohort studied by Webb and colleagues in South Africa, the laboratory tests that were most frequently abnormal as compared to the local normal range were CRP (100%), sodium (98%), ferritin (98%), D-Dimer (98%), haemoglobin (88%) and neutrophil count (88%)(15). In the work by Feldstein and colleagues, patients with MIS-C had higher neutrophil to lymphocyte ratio (median, 6·4 vs 2·7, p< .001), higher C-reactive protein level (median, 152mg/L), and lower platelet count (16).

As regards cardiac evaluation in our cohort, 4 patients had myocardial dysfunction, 3 had valvulitis and 1 had pericarditis. There was no documentation of any further follow up except for 1 child who had normal cardiac evaluation at six weeks post discharge. On the contrary, in the South African cohort, 71% had cardiac involvement which included pericardial effusions (17·6%), mitral regurgitation (36·8%) and coronary artery aneurysms (5·9%) (15). The estimated median ejection fraction was 47% (IQR 39,60)(15). Children with MIS-C and hypotension had a lower EF compared to non-hypotensive patients(15). In the cohort studied by Fieldstein and co-workers, the most severe cardiovascular involvement from MIS-C, included left ventricular dysfunction and coronary artery aneurysms which resolved within 30 days (16).

In our study treatments offered to the MIS-C patients included; antibiotics n = 16 (80·0%), intravenous immunoglobulin (IVIG) n = 2 (10·0%), systemic steroids n = 3 (15·0%) and non-steroidal anti-inflammatory drugs (NSAIDS) n = 5 (25·0%). Among the patients in the South African cohort, IVIG was the most frequently used medication given to 94·1 % of the children (15). Other medications given were intravenous methylprednisolone given to 64·7% of the children and 6% (4/68) of the children received an IL-6 inhibitor (tocilizumab) (15). In our cohort, patients responded well to IVIG and steroid therapy with no requirement of biological therapies. Nonetheless, these therapies are till beyond the reach of many children who need it due to cost and the monitoring required during their administration.

Some of the challenges faced in management and conducting research among MIS-C cases in our context is lack of resources for diagnostics and management. One in every 2 children did not have any COVID test done. In our cohort a quarter of the participants had a COVID-19 PCR test done. This low proportion could be attributed to low index of suspicion of COVID, lack of availability of the investigations or the high cost of the tests. Similarly, Webb et al summarized the first 23 cases of MIS-C treated at The Red Cross War Memorial Children’s Hospital and Tygerberg Children’s Hospital, Cape Town, South Africa, from June 4 to July 24, 2020(13). Proving previous COVID-19 disease (or SARS-CoV-2 infection), or likely contact with someone who has had COVID-19, was a limitation in their data because of poor access to SARS-CoV-2 antibody testing and restricted community testing in the region (13). Most of this cohort had no confirmed or suspected infection or no contact with COVID-19, and no access to antibody tests, but all met clinical diagnostic criteria and had likely community contact with the disease (13). A high level of suspicion was required during diagnosis because the presenting features of MIS-C were nonspecific (persistent fever, rash, and abdominal pain) (13). Our case series found that none of our patients had any known potential risk factor other than COVID-19 contact.

In our cohort, among the patients admitted to the critical care unit, compensated cardio-respiratory failure was a common feature. Webb et al found 12 (52%) of the 23 children required admission to an intensive care unit, most commonly due to myocardial dysfunction (13). Work done by Feldstein and colleagues illustrated that compared to patients with COVID-19, patients with MIS-C were more likely to be 6 to 12 years old (40·8%vs 19·4%) and non-Hispanic Black (32·3%vs 21·5%)(17). This features are similar to the demographic characteristics of our cohort. It is postulated that the cardiovascular dysfunction is likely due to severe systemic inflammation and acute stress more often than from ischemia or direct virus-mediated myocardial damage(18). On the contrary, Matsubara et al demonstrated persistent abnormalities in strain and diastolic function in patients with MIS-C and normal ejection fraction (19). These data, together with literature in adult patients with COVID-19, suggest that subclinical myocardial injury may persist even when traditional measures of left ventricular systolic function are normal (18). Despite this, only 30% (6 of 20) of our cases had a cardiac assessment further highlighting the gap in clinical are for these patients.

In order to understand the long-term implications for myocardial health, including risk for myocardial fibrosis and diastolic dysfunction, it is critical to have comprehensive assessment of left ventricular systolic and diastolic function in a large, multicenter cohort followed up longitudinally with centralized review of cardiac imaging (18). Cardiac magnetic resonance imaging and the rare endomyocardial biopsy or postmortem specimen may further help to clarify the underlying pathology and mechanisms of myocardial involvement in MIS-C (18). Feldstein and colleagues showed among patients with MIS-C with reduced left ventricular systolic function (172/503, 34·2%) and coronary artery aneurysm (57/424, 13·4%), an estimated 91·0% (95%CI, 86·0%−94·7%) and 79·1 % (95%CI, 67·1 %−89·1 %), respectively, normalized within 30 days (16). Future studies using standardized protocols and core laboratory interpretation will build on the results of this study. In our study, only 1 patient had a repeat cardiac evaluation 6 weeks after diagnosis.

Most patients in our case series did not experience severe respiratory symptoms. It is most likely that most patients may have had COVID-19 several weeks’ prior or were completely asymptomatic in the respiratory system. Due to the various cycles of the pandemic surge, it is postulated that we shall see more cases of MIS-C. We encourage child health professionals to collaborate locally, regionally and internationally in carrying out comparative effectiveness research to determine the most appropriate and feasible treatment modalities and seek biomarkers and other predictors to identify children at high risk of MIS-C and intervene promptly and appropriately to mitigate against premature morbidity and mortality.

Our study had several limitations. First, data collection was retrospective thus there were cases of missing data or incomplete reporting. We mitigated this by training the research personnel at each site that participated in data collection and recording. Second, missing data were not imputed and might be non-random. Third, participating hospitals may not be generalizable and likely overrepresented patients seeking care at tertiary care centers. Fourth, only 20% of patients had echocardiograms, most patients did not have detailed cardiac assessments. Consequently, left ventricular dysfunction and coronary aneurysms could have been underappreciated. Fifth, the efficacies of different immunomodulatory regimens on recovery of cardiac function in the current study were not examined. Sixth, because MIS-C is thought to be delayed in onset after SARS-CoV-2 infection, its distinction from acute COVID-19 could be improved by elucidating the temporal progression from viral exposure to disease onset which we were unable to ascertain given the retrospective nature of the study. Lastly, due to lack of availability of resources, we did not have the capacity to follow up diligently the 47 county referral hospitals to encourage them to enroll participants.

In conclusion, we provide a detailed clinical description of a cohort of children with confirmed and suspected MIS-C cases in Kenya. These data demonstrate that MIS-C occurs in our setting, and can cause serious disease in the paediatric population in our region. Lack of access to care means that the rates in this study are likely lower than the cases nationally. Further studies should be done to discover true case ascertainment, and highlights the case for equity in vaccination efforts for children and adolescents.

## Figures and Tables

**Table 1:** Clinical feature of MIS-C Patients

Clinical Features		Total					
		N = 20		Confirmed MISC*		Suspected MISC	
Fever	Yes	18	90.0%	4	66.7%	14	100.0%
	No	2	10.0%	2	33.3%	0	0.0%
Rash	Yes	12	60.0%	2	33.3%	10	71.4%
	No	8	40.0%	4	66.7%	4	28.6%
Bilateral Non-Purulent Conjunctivitis	Yes	4	20.0%	1	16.7%	3	21.4%
	No	14	70.0%	4	66.7%	10	71.4%
	Unknown	2	10.0%	1	16.7%	1	7.1%
Oral Mucosal Inflammation Signs	Yes	13	65.0%	2	33.3%	11	78.6%
	No	6	30.0%	3	50.0%	3	21.4%
	Unknown	1	5.0%	1	16.7%	0	0.0%
Peripheral Cutaneous Inflammation Signs	Yes	10	50.0%	1	25.0%	9	64.3%
	No	6	30.0%	2	50.0%	4	28.6%
	Unknown	4	20.0%	1	25.0%	1	7.1%
Hypotension	Yes	2	10.0%	1	16.7%	1	7.7%
	No	11	55.0%	3	50.0%	8	61.5%
	Unknown	7	35.0%	2	33.3%	4	30.8%
Tachycardia	Yes	16	80.0%	3	50.0%	13	100.0%
	No	3	15.0%	3	50.0%	0	0.0%
	Unknown	1	5.0%	0	0.0%	0	0.0%
Prolonged capillary refill Time	Yes	16	80.0%	5%	83.3%	11	84.6%
	No	3	15.0%	1	16.7%	2	15.4%
	Unknown	1	5.0%	0	0.0%	0	0.0%
Pale / Mottled Skin	Yes	1	5.0%	1	16.7%	0	0.0%
	No	17	85.0%	4	66.7%	13	92.9%
	Unknown	2	10.0%	1	16.7%	1	7.1%
Cold Hands / Feet	Yes	1	5.0%	1	20.0%	0	0.0%
	No	15	75.0%	3	60.0%	12	85.7%
	Unknown	4	20.0%	1	20.0%	2	14.3%
Urinary Output < 2 mL/kg/hr	No	3	15.0%	0	0.0%	3	23.1 %
	Unknown	17	85.0%	5	100.0%	10	76.9%
Chest Pain	Yes	1	5.0%	0	0.0%	1	7.1%
	No	17	85.0%	5	83.3%	12	85.7%
	Unknown	2	10.0%	1	16.7%	1	7.1%
Tachypnoea (age-appropriate)	Yes	1	5.0%	0	0.0%	1	7.7%
	No	18	90.0%	6	100.0%	12	92.3%
	Unknown	1	5.0%	0	0.0%	0	0.0%
Respiratory distress	Yes	1	5.0%	0	0.0%	1	7.1%
	No	19	95.0%	6	100.0%	13	92.9%
Abdominal pain	Yes	10	50.0%	3	50.0%	7	50.0%
	No	9	45.0%	3	50.0%	6	42.9%
	Unknown	1	5.0%	0	0.0%	1	7.1%
Diarrhoea	Yes	10	50.0%	3	50.0%	7	50.0%
	No	10	50.0%	3	50.0%	7	50.0%
Vomiting	Yes	15	75.0%	3	50.0%	12	85.7%
	No	5	25.0%	3	50.0%	2	14.3%

* Confirmed is either COVID Positive test or Positive COVID contact within Household

**Table 2:** Laboratory features of MIS-C Patients

Laboratory Parameter									
		Total		Confirmed MISC Cases *			Suspected MISC Cases		
	Median	IQR		Median	IQR		Median	IQR	
Hemoglobin (g/L) (n = 8)	10.6	8.9	11.35	9.2	7.25	10.85	11.1	10.3	12.3
Total WBC count (×109/L) (n = 8)	13.77	10.24	21.16	16.73	10.52	21.79	13.49	8.01	18.2
Neutrophils (×109/L) (n = 7)	7.85	3.5	11.2	11.2	3.5	18.85	7.61	4.83	8.14
Lymphocytes (×109/L) (n = 7)	6.29	2.14	8	6.59	2.14	8	4.93	2.63	7.95
Platelets (×109/L) (n = 6)	490.5	366	592	473	366	580	496.5	303	860.5
INR (n = 1)	1.3	1.3	1.3	1.29	1.29	1.29	.	.	.
CRP (mg/L) (n = 7)	32	4	74	4	4	32	65.46	31.96	93.5
Ferritin (ng/mL) (n = 3)	369.8	77.13	894.65	894.65	894.65	894.65	223.47	77.13	369.8
Sodium (mEq/L) (n = 6)	137	135	140	136.5	135	138	138	134	141

* Confirmed is either COVID positive test or positive COVID contact within Household

## Data Availability

All de-identified data will be available on reasonable request to the corresponding author within a reasonable timeframe.
